# The two faces of tropospheric ozone: a harmful yet essential constituent

**DOI:** 10.1093/nsr/nwaf491

**Published:** 2025-11-07

**Authors:** Xuefei Ma, Qindan Zhu, Zhaofeng Tan, Keding Lu, Ke Li, Xiao Lu, Haichao Wang, Yuanhang Zhang

**Affiliations:** State Key Laboratory of Regional Environment and Sustainability, State Environmental Key Lab for Ozone Pollution Control, College of Environmental Sciences and Engineering, Peking University, China; Department of Earth, Atmospheric and Planetary Sciences, Massachusetts Institute of Technology, USA; State Key Laboratory of Regional Environment and Sustainability, State Environmental Key Lab for Ozone Pollution Control, College of Environmental Sciences and Engineering, Peking University, China; State Key Laboratory of Regional Environment and Sustainability, State Environmental Key Lab for Ozone Pollution Control, College of Environmental Sciences and Engineering, Peking University, China; Collaborative Innovation Center of Atmospheric Environment and Equipment Technology, Jiangsu Key Laboratory of Atmospheric Environment Monitoring and Pollution Control, Joint International Research Laboratory of Climate and Environment Change, School of Environmental Science and Engineering, Nanjing University of Information Science & Technology, China; Southern Marine Science and Engineering Guangdong Laboratory (Zhuhai), School of Atmospheric Sciences, Sun Yat-sen University, China; Southern Marine Science and Engineering Guangdong Laboratory (Zhuhai), School of Atmospheric Sciences, Sun Yat-sen University, China; State Key Laboratory of Regional Environment and Sustainability, State Environmental Key Lab for Ozone Pollution Control, College of Environmental Sciences and Engineering, Peking University, China

**Figure 1.**
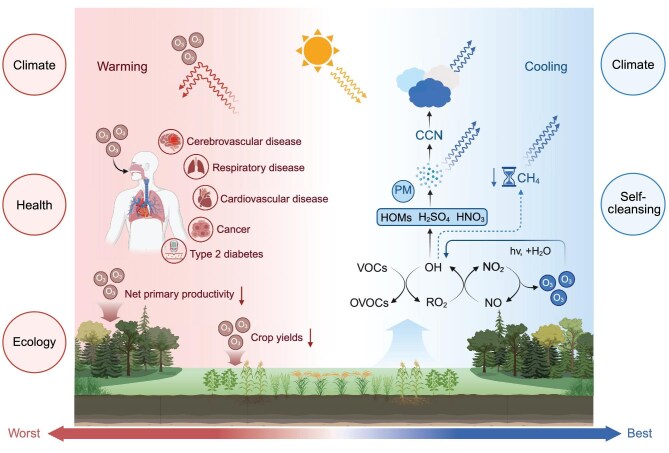
Schematic diagram of tropospheric ozone impacts on human health, ecosystem, and climate. The left panel illustrates ozone’s detrimental effects on human health and ecosystems, along with its direct radiative forcing contributing to global warming. The right panel demonstrates ozone’s indirect cooling effects through chemical feedback mechanisms that influence other short-lived climate forcers, including methane lifetime reduction, scattering aerosol particle formation, and cloud condensation nuclei activation. Abbreviations: PM, particulate matters; HOMs, highly oxidized molecules; OVOCs, oxygenated volatile organic compounds. Arrows indicate key process linkages, with color coding distinguishing warming (red) and cooling (blue) pathways.

Ozone plays two starkly distinct roles in the atmosphere. In the stratosphere, it forms a critical protective shield against ultraviolet radiation, safeguarding life on Earth, whereas in the troposphere, it presents a paradoxical duality by acting as both a harmful ‘super pollutant’ and an essential atmospheric oxidant (Fig. 1).

As a harmful ‘super pollutant’, tropospheric ozone poses significant threats to public health, climate change, and food security. Epidemiological studies have established a clear link between elevated ambient ozone exposure and a range of adverse health effects, including impaired lung function, heightened oxidative stress, systemic inflammation, and elevated risks of cardiovascular and respiratory diseases. According to the Global Burden of Disease study, approximately half a million annual premature deaths are attributable to ozone exposure, primarily due to respiratory causes [1]. A meta-analysis of all-cause mortality suggests that the actual health burden may be considerably higher [2]. Notably, significant health risks persist even at ozone concentrations that fall below previous regulatory standards. Moreover, ozone frequently coexists with other environmental stressors, such as PM_2.5_ and high temperature, and their synergistic interactions, which are not well acknowledged, can amplify health impacts beyond those of ozone alone. This growing body of evidence prompted the World Health Organization (WHO) to substantially tighten its Air Quality Guidelines in 2021, lowering the maximum daily 8-hour average to below 100 μg/m^3^ and introducing a new peak-season average limit of below 60 μg/m^3^ for long-term risk management.

As the third most potent greenhouse gas, tropospheric ozone has been significantly elevated by human activities to concentrations well above pre-industrial levels and has accordingly driven ∼0.23°C of global warming [3]. Its near-term climate impact is intensifying due to rising concentrations and its properties as a short-lived climate forcer. Moreover, ozone disrupts ecosystems by inducing oxidative stress in plants upon stomatal uptake, impairing critical physiological functions like photosynthesis and nutrient transport. This reduces yields of staple crops like wheat, soybean, rice, and maize by up to 26% and diminishes forest net primary productivity by up to 11% [4]. The resulting decline in carbon sequestration capacity can further amplify global warming through carbon cycle feedbacks. Reducing anthropogenic emissions of ozone precursors is therefore an urgent and unequivocal priority for protecting health and ecosystems in the coming decades. Yet, tropospheric ozone is essential as the primary precursor of hydroxyl radicals (OHs), which act as a natural atmospheric ‘detergent’ by oxidizing and removing reduced pollutants, a process that maintains the atmosphere’s self-cleansing capacity thus avoiding overaccumulation of harmful pollutants [5]. This inherent duality creates complex policy trade-offs, necessitating comprehensive understanding and integrated strategies that balance air quality improvement with climate change mitigation.

Tropospheric ozone is primarily produced through complex photochemical reactions involving precursors such as NO_x_, CO, CH_4_, and volatile organic compounds (VOCs) in the presence of sunlight. Given its short chemical lifetime, typically on the order of days to weeks, tropospheric ozone exhibits pronounced spatiotemporal variations that depend on precursor emissions, meteorological conditions, and geograpical environment. High ozone events typically develop under specific synoptic patterns, such as subtropical high-pressure systems and stationary fronts, which foster ozone formation through persistent strong solar radiation and further promote accumulation by inducing atmospheric stagnation and strong temperature inversions that trap pollutants near the surface. These large-scale meteorological influences are often intensified by mesoscale circulations, including sea breezes and mountain-valley winds. In regions with intensive anthropogenic emissions, such as eastern China, the western United States, and southern Europe, the interplay between emissions and these multi-scale meteorological processes frequently leads to severe ozone pollution. The coupling also shapes distinctive vertical ozone distribution. A characteristic pattern involves near-surface ozone concentrations being suppressed by fresh NO emissions in urban cores, while ozone accumulates in the lower free troposphere beneath the elevated inversion layers established by synoptic systems. These distribution patterns show strong seasonal and diurnal cycles, with the highest ozone levels generally occurring during warm seasons and around midday when solar radiation is at its most intense. In addition, weather systems like low-level jets facilitate the long-range transport of ozone and its precursors, further enhancing the spatial heterogeneity of tropospheric ozone concentrations across different regions.

The nonlinear relationship between ozone and its precursors presents a further challenge for pollution control [6]. During the 2020 COVID-19 lockdowns in China, for example, sharp and unintended emission reductions, which were characterized by a more pronounced decline in NO_x_ than VOCs, led in some regions to a paradoxical increase in near-surface ozone and an enhancement of atmospheric oxidizing capacity, which in turn exacerbated secondary aerosol formation [7]. Additional complexity arises from the intricate interactions between ozone and particulate matter, particularly the synergistic effects on secondary aerosol production and the antagonistic effect resulting from heterogeneous HO_2_ uptake on aerosol surfaces [8]. Given the intertwined challenges posed by nonlinear chemical processes and heterogeneous spatial distribution, elucidating the scientific mechanisms governing ozone behavior remains an urgent priority for developing effective control policies.

Tropospheric ozone has exerted a radiative forcing of 0.2–0.4 W/m^2^ since pre-industrial times, ranking it as the third-most significant greenhouse gas after CO_2_ and CH_4_, though considerable uncertainties persist in these estimates [3]. First, the radiative forcing attributable to tropospheric ozone exhibits particularly large uncertainties due to pronounced heterogeneity in both horizontal and vertical distributions, which is a unique characteristic among greenhouse gases that stems from its photochemical production and oxidative nature. Second, direct measurements of tropospheric ozone are only available for the recent decades, with no observational records of ozone concentration levels prior to 1850. Historical reconstructions rely on proxy data, such as the clumped-isotope composition of oxygen (^18^O^18^O in O_2_) from polar firn and ice cores, combined with atmospheric chemistry model simulations, which suggest a global tropospheric ozone increase of <40% between 1850 and 2005, with most of the rise occurring from 1950 to 1980 [9]. However, such reconstructions are subject to considerable uncertainties stemming from the indirect nature of isotopic proxies, the limited spatial representativeness of polar ice cores, and imprecise pre-industrial emission inventories. This uncertainty is reflected in the significant divergence among model simulations, particularly for the pre-industrial era, which cannot be robustly validated due to the absence of direct observations [10]. Finally, model-dependent assumptions embedded in the calculation of ozone radiative forcing, such as the common simplification that equates ozone’s effective radiative forcing to its stratospheric-temperature–adjusted radiative forcing, inherently overlook tropospheric adjustments and thereby amplify the overall uncertainties.

Beyond its direct radiative effects, tropospheric ozone plays a critical role in indirect climate forcing through complex atmospheric chemistry interactions. As the dominant source of OH, ozone governs atmospheric oxidation capacity, which in turn determines the atmospheric lifetimes of potent greenhouse gases like CH_4_ and hydrofluorocarbons (HFCs). This interdependence creates important trade-offs in emission reduction strategies. For instance, while reducing NO_x_ emissions improves air quality by decreasing ozone pollution, it may also suppress OH concentrations, thereby prolonging methane’s atmospheric lifetime and potentially offsetting climate benefits. The COVID-19 pandemic provided compelling observational evidence of this linkage, where a modest 1.6% reduction in OH concentrations, predominately due to decreased NO_x_ emissions and associated lower tropospheric ozone, resulted in a dramatic 53% increase in the methane growth rate anomaly [11], underscoring the rapid radiative forcing responses mediated by ozone-driven photochemistry. Furthermore, through these same photochemical oxidation processes, ozone and its derived OH radicals drive the formation of secondary aerosols, including sulfates, nitrates, and organic compounds, thereby linking ozone chemistry directly to aerosol-associated climate effects [12]. These aerosols influence climate through dual mechanisms: direct scattering and absorption of solar radiation (particularly the scattering effect of sulfate particles) and indirect cloud modification via cloud condensation nuclei (CCN) activation. The cloud-mediated effects represent a particularly significant climate forcing pathway, in which increased CCN concentrations elevate cloud droplet number concentration, liquid water paths, and albedo. These microphysical enhancements collectively intensify the scattering of solar radiation, resulting in a substantial negative radiative forcing and a net cooling effect on the climate system. However, the current understanding of these ozone-mediated climate pathways remains incomplete. The complex ozone-aerosol-cloud interactions constitute the largest uncertainty in radiative forcing assessments, and their continued inadequate representation in climate models potentially introduces substantial biases in climate projections.

To address critical knowledge gaps in the interactions between tropospheric ozone and Earth systems, we propose to prioritize research efforts in several key areas: (1) developing multi-scale monitoring networks that synergistically integrate ground-based stations, satellite remote sensing, and airborne platforms, coupled with advanced data assimilation techniques, to map high-resolution, three-dimensional ozone and precursor distributions across urban-to-continental scales and resolve critical spatial gradients associated with boundary layer processes and free tropospheric transport; (2) advancing pre-industrial ozone reconstructions through synergistic development of novel multi-isotope proxies for historical atmospheric oxidation state constraints, enhanced Earth system modeling with data assimilation for generating physically consistent global ozone fields, and rigorous validation against modern analog observations from pristine regions to benchmark model performance under a low-emission scenario, thereby reducing radiative forcing uncertainties and establishing robust long-term ozone trend analyses; (3) advancing process-level understanding of ozone-aerosol-cloud interactions through targeted chamber experiments and observationally constrained modelling, with particular emphasis on improving the representation of fundamental processes such as online-coupled chemistry-aerosol-cloud interactions, ozone-driven aerosol aging impacts on secondary organic aerosol properties, and composition-dependent cloud activation in order to reduce uncertainties in simulating nonlinear chemical feedbacks mediated by ozone and OH within global climate models; (4) formulating robust science-based policy frameworks for short-lived climate forcers through the development of advanced, fully coupled chemistry-climate models capable of simulating ozone-methane-aerosol co-evolution pathways across diverse emission scenarios, with subsequent translation of quantified trade-offs into decision-making metrics to identify synergistic mitigation strategies that maximize climate and air quality co-benefits while minimizing unintended consequences; and (5) improving health impact assessments for ozone by integrating molecular-level mechanistic studies to clarify the pathways from respiratory ozone exposure to systemic effects and bridge the gap with epidemiological research, and by a coordinated effort to investigate the synergistic effects of co-exposure with other environmental stressors, evaluate the role of ozone-derived oxidation products, and verify low-concentration dose-response relationships through toxicological experiments, thereby providing critical evidence for precision health risk assessment and science-based air quality standard refinement.

These research imperatives underscore the need for policy solutions that simultaneously address near-term air quality goals and long-term climate targets, requiring careful optimization of ozone reduction strategies to protect human health and ecosystems while maintaining its critical atmospheric oxidation capacity. Achieving this balance demands unprecedented interdisciplinary collaboration, robust observational investments, and coordinated international governance, a scientific and policy challenge of pressing global importance.
